# Supplementary material for: WordCommentsAnalyzer: A windows software tool for qualitative research
Source: F1000Res. 2018 Sep 4;7:536. Originally published 2018 May 3. [Version 2] doi: 10.12688/f1000research.14819.2 (PMC6137411; doi:10.12688/f1000research.14819.2)
Supplement: Supplementary file 3 [file f1000research-7-17614-s0002.tgz › b2a79496-4ada-47c4-9682-a99ce867836d.zip!/Eight Iranian Health Journals/Journal of Health System Research _9.docx]

© Journal of Health System Research . This work is licensed under CC BY-NC 4.0 (https://creativecommons.org/licenses/by-nc/4.0/).

Volume & Issue: دوره 12، شماره 1، 1395

Title: بررسی میزان آگاهی دانش‌آموزان دختر دبیرستانی شهر آشخانه در زمینه ایدز در سال 1391: کاربردی از مدل اعتقاد بهداشتی

http://hsr.mui.ac.ir/index.php/jhsr/article/view/1992

مقدمه: مطالعه حاضر با هدف تعیین میزان آگاهی دانش آموزان دختر دبیرستانی شهر آشخانه با بکارگیری مدل اعتقاد بهداشتی در سال 1391 انجام گرفت. روش‌ها: این مطالعه به صورت مقطعی و با شرکت 384 دانش‌آموز دبیرستانی انجام شد. ابزار جمع‌آوری داده‌ها، پرسش‌نامه‌ای مشتمل بر سازه‌های مدل اعتقاد بهداشتی بود که روایی و پایایی آن تأیید گردید. در نهایت، داده‌های به دست آمده با استفاده از آزمون‌های Independent t و ANOVA در نرم‌افزار SPSS مورد تجزیه و تحلیل قرار گرفت. یافته‌ها: پس از تقسیم‌بندی آگاهی دانش‌آموزان مشخص شد که 66 نفر (2/17 درصد) آگاهی خوب، 303 نفر (9/78 درصد) آگاهی متوسط و 15 نفر (9/3 درصد) آگاهی ضعیفی داشتند. میانگین سازه‌های مدل اعتقاد بهداشتی نشان داد که میانگین حساسیت درک شده 04/13 ± 48/51، شدت درک شده 97/18 ± 90/51، منافع درک شده 60/23 ± 90/72 و موانع درک شده 00/21 ± 74/53 بود. نتیجه‌گیری: نتایج مطالعه حاضر حاکی از آن بود که آگاهی دانش‌آموزان در حد متوسط است و آموزش مبتنی بر مدل اعتقاد بهداشتی و تأکید بر سازه حساسیت درک شده در این دانش‌آموزان ضروری به نظر می‌رسد.

Title: پيش‏بيني سطح سلامت روان دانشجويان دانشگاه علوم پزشكي اصفهان بر اساس متغیرهای جمعیت ‏شناختی

http://hsr.mui.ac.ir/index.php/jhsr/article/view/2406

مقدمه: دانشجویان رشته‏های علوم پزشکی به دلیل مواجهه با محیط پرتنش و وجود عوامل تنش‏زا در تجارب آموزشی و بالینی، از نظر ابتلا به اختلالات روانی جزء گروه‏های پرخطر محسوب می‏شوند. با توجه به تأثیر سلامت روان بر عملکرد فرد، مطالعه حاضر با هدف پيش‏بيني سطح سلامت روان دانشجويان دانشگاه علوم پزشكي اصفهان بر اساس متغیرهای جمعیت ‏شناختی انجام شد. روش‌ها: در این مطالعه توصیفی- همبستگی، 521 نفر از دانشجویان دانشگاه علوم پزشکی اصفهان شرکت نمودند که با روش نمونه‌گيري تصادفي طبـقه‌اي وارد مطالعه شدند. ابزار جمع‌آوری اطلاعات، فرم اطلاعات دموگرافیک و پرسش‌نامه سلامت عمومی 28 سؤالی (General Health Questionnaire یا GHQ-28) بود. داده‌ها پس از جمع‌آوری با استفاده از آمار توصیفی و آزمون‌های Independent t، ANOVA و تحلیل رگرسیون خطی چندگانه در نرم‌افزار SPSS مورد تجزیه ‌و تحلیل قرار گرفت. یافته‌ها: بر اساس نتایج تحلیل رگرسـیون، جنسیت با ضریب تعیین 6/9 درصد، بیشترین سهم را در پیش‏بینی سلامت روان دانشجویان ایفا ‏کرد (میانگین نمره اختلال سلامت روان در دختران بیشتر از پسران بود) و متغیرهای جنسیت، تأهل، وضعیت سکونت، علاقمندی به رشته، شاخص توده بدنی، شغل، وضعیت اقتصادی، استعمال سیگار و تحصیلات والدین نیز در مجموع، 1/30 درصد از واریانس سلامت روان دانشجویان را تبیین نمود (1/30 = R2). نتیجه‌گیری: با توجه به نتایج مطالعه حاضر، نزدیک به نیمی از دانشجویان مورد مطالعه نمره بالاتر از نقطه برش را کسب کردند و می‏توان اذعان داشت که سلامت روان آنان در سطح پایینی قرار دارد. همچنین، عوامل جمعیت ‏شناختی مؤثر بر سلامت روان دانشجویان قابل توجه بود. بنابراین، برنامه‏ریزی به منظور شناسایی دقیق عوامل مؤثر بر سلامت روان دانشجویان و کمک به ارتقای آن ضروری به نظر می‌رسد.

Title: بررسي حذف فلوراید از آب آشامیدنی با بوکسیت معدن مندون: مطالعه موردی آب منطقه کوهبنان

http://hsr.mui.ac.ir/index.php/jhsr/article/view/2052

مقدمه: غلظت فلوراید آب آشامیدنی یکی از شاخص‌های مؤثر در سلامت انسان است. بنابراین، یافتن راهی برای حذف فلوراید بیش از مقدار استاندارد، از اهداف مهم در پروژه‌های تأمین آب می‌باشد. این مطالعه با هدف، بررسی کارایی بوکسیت معدن مندون در حذف فلوراید آب آشامیدنی انجام گرفت. روش‌ها: مطالعه حاضر از نوع تجربی بود که از اردیبهشت تا آبان سال 1392 بر روی نمونه‌های سنتتیک و واقعی آب منطقه کوهبنان در مرکز تحقیقات بهداشت محیط دانشگاه علوم پزشکی کرمان انجام شد. بوکسیت از معدن مندون استان کهگیلویه و بویراحمد تهیه گردید و تجزیه و تحلیل آن به روش طیف‌سنجی فلورسانس اشعه ایکس و طیف‌سنجی تفرق اشعه ایکس انجام گرفت. تأثیر پارامترهای pH، زمان تماس، مقدار جاذب و غلظت‌های مختلف فلوراید بر روی حذف، با استفاده از محلول‌های سنتتیک بررسی گردید و شرایط بهینه روی نمونه واقعی انجام شد. به منظور درک بهتر فرایند جذب، سنتتیک و ایزوترم جذب نیز مورد بررسی قرار گرفت. میزان فلوراید به روش اسپادنس تعیین و در نهایت، داده‌ها در نرم‌افزار Excel تجزیه و تحلیل شد. یافته‌ها: حداکثر راندمان حذف نمونه سنتتیک در 7 = pH، زمان تماس 120 دقیقه توسط محلول 20 گرم بوکسیت در لیتر، به میزان 96/76 درصد به دست آمد. راندمان حذف در نمونه واقعی و در شرایط بهینه، 64/56 درصد بود. یافته‌ها نشان داد که جذب فلوراید، از ایزوترم Langmuir با ضریب همبستگی 719/0 و سنتتیک واکنش درجه دوم با ضریب همبستگی 989/0 پیروی نمود. نتیجه‌گیری: بوکسیت مندون بدون نیاز به آماده‌سازی اولیه جهت حذف فلوراید از آب‌های آشامیدنی، دارای راندمان جذب قابل قبولی می‌باشد.

Title: ارتباط اعتیاد به اینترنت با خودکارامدی اجتماعی، سبک‌های هویت و تفاوت‌های جنسیتی در دانشجویان

http://hsr.mui.ac.ir/index.php/jhsr/article/view/2341

مقدمه: با وجود تمام ویژگی‌هاي مثبت اینترنت، اما نگرانی‌هاي جدی در خصوص استفاده و تأثیرات این تکنولوژی بر سلامت‌ روانی انسان وجود دارد. پژوهش حاضر با هدف تعیین ارتباط اعتیاد به اینترنت با خودکارامدی ‌اجتماعی، سبک‌هاي هویت و تفاوت‌هاي جنسیتی در دانشجویان انجام گرفت. روش‌ها: در این پژوهش توصیفی- همبستگی، 200 نفر دانشجوی مقطع کارشناسی و کارشناسی ارشد دانشگاه خوارزمی به روش نمونه‌گیری در دسترس انتخاب شدند و پرسش‌نامه‌های اعتیاد به اینترنت Young، خودکارامدی ‌اجتماعی Smith‏ و ‏Betz و سبک‌هاي هویت Berzonsky را تکمیل نمودند. داده‌ها با استفاده از آزمون‌هاي Independent t، ANOVA، ضریب همبستگی Pearson و تحلیل رگرسیون چند متغیره در نرم‌افزار SPSS مورد تجزیه و تحلیل قرار گرفت. یافته‌ها: اعتیاد به اینترنت با خودکارامدی‌ اجتماعی (050/0 > P) و سبک‌های هویت اطلاعاتی و هنجاری رابطه منفی (010/0 > P) و با سبک هویت سردرگم- اجتنابی رابطه مثبتی داشت (050/0 > P). تفاوت‌هاي جنسیتی در میزان استفاده از اینترنت مؤثر و این میزان در پسران بالاتر از دختران بود (010/0 > P). همچنین، 23 درصد از کل واریانس اعتیاد به اینترنت توسط متغیرهای مذکور تبیین گردید (010/0 > P). نتیجه‌گیری: فضای مجازی بسیار جذاب و جالب است و نوجوانان و جوانان بیشتر از سایر افراد به استفاده از آن روی می‌آورند. بنابراین، والدین و مربیان باید توجه خاصی به رشد ذهنی و اجتماعی این قشر به خصوص در مرحله‌ بحران هویت داشته باشند و با ارایه روش تربیتی مناسب، زمینه رشد و تکامل را برای فرزندان خود فراهم نمایند تا از زندگی سالمی بهره‌مند شوند.

Title: بررسی میزان تحقق استانداردهای محیط ایمن در بیمارستان‌های منتخب دانشگاه علوم پزشکی شیراز

http://hsr.mui.ac.ir/index.php/jhsr/article/view/1920

مقدمه: به علت وجود شرايط خاص در بيمارستان از نظر تجمع افراد، حضور افراد ناتوان و بيمار، وجود تجهيزات مختلف و پيچيده، مواد شيميايي و عفوني مصرفي، مواد قابل اشتعال، مواد شيميايي سرطان‌زا و تشعشعات يونيزان، رعايت اصول ايمني جهت پیشگیری از رخداد حوادثي همچون آتش‌سوزي و برق‌ گرفتگي و مواجهه با مقادير غير مجاز عوامل مخاطره‌آميز در بیمارستان‌ها ضروری است. اين مطالعه با هدف بررسی، میزان تحقق استانداردهای مربوط به ایمنی محیط بر اساس استاندارد بیمارستان‌های دوستدار ایمنی، انجام شد. روش‌ها: مطالعه توصيفی- مقطعی حاضر در شش بيمارستان آموزشی شهر شيراز صورت گرفت. وضیعت ایمنی بیمارستان‌ها بر اساس راهنمای سازمان جهانی بهداشت تحت عنوان بیمارستان‌های دوستدار ایمنی، بررسی شد. بدین منظور، چک‌ليست‌های مطالعه به شیوه بررسی میدانی تکميل و امتیازبندی گردید و مورد تجزیه و تحليل قرار گرفت. یافته‌ها: میانگین درصد تحقق استانداردهای الزامی و اساسی در کل بیمارستان‌های مورد بررسی به ترتیب 100 و 3/78 درصد بود. میانگین درصد تحقق در زیرگروه‌های استانداردهای اساسی شامل 1D و 2D نیز به ترتيب 7/76 و 6/89 درصد به دست آمد. نتیجه‌گیری: نتايج مطالعه حاضر نشان داد که همه بیمارستان‌های مورد بررسی از نظر استانداردهای ایمنی محيط، در سطح 2 طبقه‌بندی می‌شوند. جهت تحقق کامل مفاد مندرج در استاندارد‌های لازم، باید اقدامات ضروری در جهت ایمن‌سازی بیمارستان‌ها به ويژه در زمينه استانداردهای اساسی گروه 1D صورت گیرد.

Title: وضعیت استفاده از طب مکمل در خانم‌های باردار مراجعه کننده به مراکز بهداشتی- درمانی شهر اصفهان در سال 1392

http://hsr.mui.ac.ir/index.php/jhsr/article/view/2027

مقدمه: استفاده از روش‌های طب مکمل در سال‌های اخیر افزایش چشمگیری به خصوص در میان زنان داشته است، تا جایی که این استفاده در بارداری نیز ادامه می‌یابد؛ در حالی که مطالعات اندکی در زمینه میزان و الگوی مصرف انجام شده است. بنابراین، با توجه به حساسیت دوران بارداری و کمبود اطلاعات در مورد نحوه به کارگیری روش‌های طب مکمل در بارداری، این مطالعه با هدف بررسی وضعیت کاربرد طب مکمل در دوران بارداری انجام گرفت. روش‌ها: مطالعه مقطعی حاضر در سال 1392 بر روی 599 نفر از خانم‌های مراجعه کننده به مراکز بهداشتی- درمانی شهر اصفهان انجام شد. اطلاعات با استفاده از مصاحبه توسط پرسشگر آموزش دیده و با کمک پرسش‌نامه جمع‌آوری گردید. داده‌های به دست آمده با استفاده از آزمون Independent t و همبستگی Pearson و Spearman در نرم‌افزار SPSS مورد تجزیه و تحلیل قرار گرفت. یافته‌ها: 8/45 درصد از مشارکت کنندگان از یک روش و 0/21 درصد از بیش از یک روش طب مکمل در بارداری استفاده نموده بودند. 2/33 درصد از خانم‌ها هیچ کدام از روش‌های‌ طب مکمل را در دوران بارداری استفاده نکرده‌ بودند. شایع‌ترین روش‌های طب مکمل به کار رفته شامل دعا و نذر، داروهای گیاهی و ماساژ بود. بین سطح تحصیلات و شغل با تعداد روش‌های طب مکمل استفاده شده در بارداری رابطه معنی‌داری وجود داشت؛ به طوری که استفاده از روش‌ها در خانم‌های شاغل (020/0 = P) و با تحصیلات بالاتر (001/0 > P) بیشتر بود. نتیجه‌گیری: نتایج مطالعه بیانگر مصرف بالای روش‌های طب مکمل در میان خانم‌های باردار می‌باشد. بنابراین، انجام تحقیقات بیشتر در مورد اثربخشی، عوارض و تداخلات هر یک از این روش‌ها پیشنهاد می‌گردد. همچنین، آموزش منسجم کارکنان بهداشت و درمان و آموزش عمومی در مورد اثرات و عوارض طب مکمل ضروری به نظر می‌رسد.

Title: بررسی شیوع افسردگی و تعیین همبسته‌های آن در دانشجویان دانشگاه علوم پزشکی شیراز

http://hsr.mui.ac.ir/index.php/jhsr/article/view/2051

مقدمه: بر اساس گزارش سازمان بهداشت جهاني، اختلالات رواني جزء 10 علل مهم ناتواني و از كارافتادگي در جهان است. مطالعه حاضر با هدف، بررسی شیوع افسردگی و تعیین عوامل مؤثر بر آن در دانشجويان صورت پذیرفت. روش‌ها: در اين مطالعه مقطعي، 358 نفر از دانشجويان دانشگاه علوم پزشكي شیراز داوطلبانه شرکت نمودند. ابزار جمع‌آوری داده‌ها پرسش‌نامه‌ای شامل دو قسمت ویژگی‌های دموگرافیک و پرسش‌نامه استاندارد Beck (فرم 21 سؤالی) بود. داده‌ها با استفاده از آزمون‌های آماری Spearman's correlation، Kruskal-Wallis و Mann–Whitney U و در نرم‌افزار SPSS مورد تجزیه و تحلیل قرار گرفت. یافته‌ها: میانگین (انحراف معیار) نمره افسردگی در افراد مورد مطالعه برابر با 98/10 (68/8) به دست آمد. همچنین، مشخص گردید که 162 نفر (25/45 درصد) از دانشجویان مورد مطالعه دچار درجات مختلفی از افسردگی هستند. بین نمره افسردگی با وضعیت تأهل، مقطع تحصیلی، رضایت از رشته، مصرف مواد روان‌گردان، امید به آینده شغلی، مشکل داشتن در امر ازدواج و پایبند بودن به امور مذهبی از نظر آماری رابطه وجود داشت. نتیجه‌گیری: شیوع افسردگی در دانشجویان مورد مطالعه بالا بود. از عواملی همچون فاکتورهای شخصی و تحصیلی و نوع سبک زندگی، می‌توان به عنوان عوامل مؤثر بر افسردگی نام برد. با توجه به شیوع بالای افسردگی بایستی تدابيري اتخاذ شود تا عوامل مؤثر در بروز اين اختلالات كاهش يابد.

Title: بررسي راندمان جاذب مگنتیک اکسید آهن در حذف رنگ نساجی Navy Blue CE-RN از محلول‌هاي آبي

http://hsr.mui.ac.ir/index.php/jhsr/article/view/2292

مقدمه: فاضلاب صنایع نساجی از عمده‌ترین آلاینده‌های محیط زیست محسوب می‌شود. Navy Blue CE-RN (NB) یک رنگ شیمیایی مهم و پرکاربرد در صنایع نساجی می‌باشد. با توجه به کارایی فرایند جذب در حذف رنگ، مطالعه حاضر با هدف بررسی حذف رنگ نساجی NB از محلول‌هاي آبي توسط جاذب مگنتیک اکسید آهن انجام گرفت. روش‌ها: این تحقیق بر روی فاضلاب سنتتیک با غلظت رنگ 50 میلی‌گرم بر لیتر انجام شد و تأثیر متغیرهاي مختلفی مانند زمان تماس، pH محلول و دوز جاذب مورد ارزیابی قرار گرفت. پس از فرایند حذف رنگ توسط جاذب مگنتیک اکسید آهن، برای جداسازی آن از محلول آبی، از دستگاه سانتریفوژ استفاده گردید. جهت تعیین میزان حذف رنگ، دستگاه DR-5000 و آزمایش COD (Chemical oxygen demand) مورد استفاده قرار گرفت. برای تعیین ابعاد جاذب نیز از تصویر تهیه شده با دستگاه میکروسکوپ الکترونی پویشی (Scanning electron microscope یا SEM) استفاده شد. یافته‌ها: میزان جذب رنگ NB با افزایش زمان تماس از 10 به 210 دقیقه، راندمان حذف را از 0/74 به 4/80 درصد افزایش داد. تغییرات pH محلول از محدوده اسيدي به قليايي حاکی از آن بود که بیشترین حذف در 6 = pH صورت گرفت. با افزايش جرم جاذب از 1 به 4 گرم بر لیتر، راندمان جذب رنگ از 1/73 به 7/93 درصد افزایش یافت. همچنین، طبق یافته‌های به دست آمده، راندمان حذف COD برابر با 4/78 درصد و ظرفیت جذب برابر با 8/11 میلی‌گرم بر گرم بود. نتیجه‌گیری: در این آزمایش، تأثیر متغیرهایی مانند زمان تماس، pH محلول و مقدار ماده جاذب بررسی شد و مشخص گردید که حذف رنگ در زمان تماس 210 دقیقه، 6 = pH و دوز جاذب 4 گرم بر لیتر حداکثر راندمان را دارد. همچنین، نتایج پژوهش حاضر نشان داد که جاذب مگنتیک اکسید آهن استفاده شده در تحقیق می‌تواند به عنوان روش قابل قبولی با کارایی بالا برای حذف رنگ نساجی NB به کار رود.

Title: وضعیت فعالیت فیزیکی کارکنان اداره‌های شهر همدان: کاربردی از سازه مراحل تغییر

http://hsr.mui.ac.ir/index.php/jhsr/article/view/2092

مقدمه: عدم فعالیت فیزیکی، چهارمین علل مرگ و میر در دنیا به شمار می‌رود. با توجه به افزایش کم‌تحرکی در بین کارمندان، این مطالعه با هدف بررسی وضعیت فعالیت فیزیکی کارمندان ادارات دولتی شهر همدان با بهره‌گیری از سازه مراحل تغییر مدل فرانظری، در سال 1392 انجام گردید. روش‌ها: مطالعه حاضر از نوع توصیفی- تحلیلی بود و بر روی 1200 نفر از کارمندان دولتی که به صورت نمونه‌گیری طبقه‌ای متناسب انتخاب شده بودند، انجام شد. ابزار جمع‌آوری داده‌ها، پرسش‌نامه سه قسمتی شامل ویژگی‌های دموگرافیک، پرسش‌نامه کوتاه سنجش فعالیت فیزیکی (Short questionnaire to assess health-enhancing physical activity‎ یا SQUASH) و الگوریتم پنج بخشی Marcus وForsyth  بود. داده‌ها با استفاده از آزمون‌های رگرسیون خطی و لجستیک، 2c و آنالیز واریانس در نرم‌افزار SPSS مورد تجزیه و تحلیل قرار گرفت. یافته‌ها: میانگین سنی افراد مورد مطالعه، 04/8 ± 12/38 سال بود. از نظر مراحل تغییر، نیمی از مشارکت کنندگان در مرحله آمادگی قرار داشتند. از نظر فیزیکی، به ترتیب 2/49 و 8/50 درصد افراد غیر فعال و فعال بودند. بین وضعیت فعالیت فیزیکی (فعال یا غیر فعال) با مراحل تغییر انجام فعالیت فیزیکی رابطه معنی‌داری وجود داشت. همچنین، رابطه معنی‌داری بین فعالیت فیزیکی با متغیرهای سن، جنس، سابقه‌ کار، تأهل و تحصیلات مشاهده شد (05/0 > P). تحصیلات و سابقه، قوی‌ترین پیشگویی کننده‌های فعالیت فیزیکی بودند (01/0 = R2 change، 312/0 = Adjusted R2). نتیجه‌گیری: با توجه به غیر فعال بودن حدود نیمی از کارمندان از نظر فیزیکی و مطلوب نبودن وضعیت آن‌ها از نظر شدت فعالیت (متوسط یا شدید)، نتایج مطالعه حاضر می‌تواند به برنامه‌ریزان جهت طراحی و اجرای استراتژی‌های مناسب و تئوری محور به منظور ارتقای فعالیت فیزیکی کارمندان، به ویژه در محیط‌های کاری کمک نماید.

Title: بررسی غلظت بنزوات سدیم در دوغ‌های توزیع شده در سطح عرضه شهر اصفهان

http://hsr.mui.ac.ir/index.php/jhsr/article/view/2673

مقدمه: دوغ نوشیدنی تخمیری است که مانند دیگر نوشیدنی‌ها قابلیت فساد میکروبی دارد. نگهدارنده اسید بنزوئیک و نمک آن (بنزوات سدیم) جهت کنترل فساد میکروبی در دوغ مورد استفاده قرار می‌گیرد. این در حالی است که بر اساس ضوابط سازمان غذا و دارو و سازمان ملی استاندارد ایران، استفاده از هر گونه نگهدارنده در شیر و فرآورده‌های لبنی ممنوع اعلام شده است. هدف از انجام پژوهش حاضر، تعیین میزان بنزوات سدیم در دوغ‌های توزیع شده در سطح عرضه شهر اصفهان بود. روش‌ها: در این مطالعه توصیفی- مقطعی، غلظت بنزوات سدیم در 36 نمونه دوغ تولیدی ازسه برند (A، B و C) توزیع شده در سطح عرضه شهر اصفهان در سال 1394، با استفاده از روش کروماتوگرافی مایع با کارایی بالا مورد ارزیابی قرار گرفت. تجزیه و تحلیل آماری در نرم‌افزار SPSS با استفاده از آزمون آماری ANOVA جهت مقایسه میانگین بنزوات سدیم در برندهای مختلف نمونه‌برداری شده دوغ صورت گرفت. در تمامی آزمون‌ها سطح معنی‌داری 05/0 در نظر گرفته شد. یافته‌ها: طبق بررسی انجام شده، دوغ تولیدی کارخانه فراورده لبنی با برند C بیشترین میزان بنزوات سدیم (ppm 55/0 ± 84/4) و برند A کمترین محتوای بنزوات سدیم با مقدار (ppm 11/0 ± 03/4) را دارا بود. در همه نمونه‌های دوغ، بنزوات سدیم شناسایی شد. اختلاف معنی‌داری در غلظت بنزوات سدیم اندازه‌گیری شده در سه برند مورد مطالعه مشاهده گردید (001/0 > P). نتیجه‌گیری: مشاهده غلظت پایینی از بنزوات سدیم در همه نمونه‌های دوغ مورد بررسی در مطالعه حاضر، می‌تواند به دلیل تشکیل طبیعی این ترکیب در ماست و انتقال آن به دوغ باشد. همچنین، نظر به فاصله زیاد بنزوات سدیم شناسایی شده در مطالعه حاضر با مقادیر تعیین شده در استاندارد بین‌المللی کدکس برای نوشیدنی‌های تخمیری تا مقدار mg/kg 300، تعیین حد مجاز بنزوات سدیم در دوغ بر اساس الگوی تغذیه‌ای بومی پیشنهاد می‌شود.

Title: عوامل مؤثر بر گرایش به مصرف فست‌فودها (غذاهای آماده) در نوجوانان

http://hsr.mui.ac.ir/index.php/jhsr/article/view/2066

مقدمه: مصرف غذاهای آماده توسط دانش‌آموزان دبیرستانی رو به افزایش است. این مطالعه با هدف، بررسی عوامل مرتبط با مصرف غذاهای آماده در نوجوانان شهر سنندج انجام شد. روش‌ها: در مطالعه مقطعی حاضر، 553 دانش‌آموز دبیرستان‌های شهر سنندج به صورت تصادفی انتخاب شدند. داده‌ها توسط پرسش‌نامه‌ای که روایی و پایایی آن تأیید شده بود، جمع‌آوری گردید و در نهایت، با استفاده از آزمون‌های آماری 2c و Independent t در نرم‌افزار SPSS مورد تجزیه و تحلیل قرار گرفت. یافته‌ها: 8/69 درصد افراد حداقل یک‌بار در هفته غذای آماده مصرف می‌کردند. نوع غذای آماده مصرفی در دو جنس متفاوت بود (001/0 > P). رابطه آماری معنی‌داری بین تحصیلات مادر و آگاهی نوجوانان از غذای آماده وجود داشت (050/0 > P). نتیجه‌گیری: با توجه به نقش مهم اعضای خانواده و به ویژه مادر بر انتخاب غذای نوجوانان و اثر سواد مادر در میزان آگاهی و گرایش دانش‌آموزان به مصرف غذای آماده، توجه بیشتر به ارتقای سطح آگاهی مادران می‌تواند راهکار مهمی در کاهش مصرف غذاهای آماده محسوب شود.

Title: بررسی سرولوژيك شیوع عفونت‌های HIV و هپاتیت B در معتادان وابسته به مواد مخدر و ارتباط آن با برخی عوامل خطر رفتاری

http://hsr.mui.ac.ir/index.php/jhsr/article/view/2064

مقدمه: یکی از مهم‌ترین مشکلات جوامع بشری، شیوع عفونت‌های Human immunodeficiency virus (HIV) و هپاتیت B در معتادان است. این مطالعه با هدف تعیین شیوع سرولوژيك عفونت‌های هپاتیت B و HIV و ارتباط آن با عوامل خطر رفتاری مربوط به آن، در معتادان وابسته به مواد مخدر مراجعه کننده به مرکز ترک اعتیاد زمزم انجام گرفت. روش‌ها: مطالعه توصیفی- تحلیلی حاضر، بر روی 220 نفر از معتادان تحت درمان نگهدارنده با متادون مراجعه کننده به مرکز ترک اعتیاد زمزم وابسته به مرکز بهداشت جنوب تهران، به مدت سه ماه (دی تا اسفند سال 1389) انجام شد. نمونه‌گیری به روش سرشماری صورت گرفت. شیوع سرمی هپاتیت B و HIV از طریق آزمایش (ELISA) Enzyme-linked immunosorbent assay بررسی گردید. مشخصات جمعیت‌شناختی و رفتاری شرکت کنندگان با استفاده از پرسش‌نامه محقق ساخته و مصاحبه چهره به چهره جمع‌آوری شد. در نهایت، داده‌ها با استفاده از آزمون‌های 2c و t مورد تجزیه و تحلیل قرار گرفت. یافته‌ها: میزان شیوع عفونت‌های HIV و هپاتیت B به ترتیب 8/1 و 1/4 درصد بود. بین اعتیاد تزریقی (001/0 > P)، تعداد دفعات تزریق در روز (001/0 > P) و استفاده از سوزن و سرنگ مشترک (001/0 > P) با آلودگی بهHIV  و هپاتیت B ارتباط آماری معنی‌داری وجود داشت. علاوه بر این، ارتباط معنی‌داری بین وضعیت تأهل با شیوع عفونتHIV  مشاهده شد (026/0 = P). همچنین، بین داشتن سابقه زندان با شیوع هپاتیت B (032/0 = P) رابطه معنی‌داری وجود داشت. نتیجه‌گیری: شیوع عفونت‌های HIV و هپاتیت B در معتادان مراجعه کننده به مرکز بهداشت زمزم، به ویژه در میان معتادان تزریقی بالا می‌باشد. بنابراین، مداخلات پیشگیری کننده مانند مشاوره و آموزش رایگان توسط افراد حرفه‌ای، توزیع سرنگ‌های استریل رایگان، غربالگری دقیق و منظم بیماری‌های منتقل شونده از راه خون و واکسیناسیون هپاتیت B برای مصرف کنندگان مواد مخدر توصیه می‌شود.

Title: تعیین غلظت مؤثر 50 درصد بازدارنده رشد (EC50) فلزات سنگین بر باکتری‌های موجود در رآکتور منقطع متوالی

http://hsr.mui.ac.ir/index.php/jhsr/article/view/2313

مقدمه: در بین آلاینده‌های شیمیایی موجود در فاضلاب، فلزات سنگین جزء ترکیبات مقاومی به شمار می‌روند که سمیت آن‌ها بر فرایندهای بیولوژیکی تصفیه فاضلاب و همچنین، بر انسان و محیط در مطالعات مختلفی به اثبات رسیده است. در مطالعه حاضر، غلظت مؤثر 50 درصد بازدارنده رشد (% Effective concentration50 یا 50EC) فلزات کادمیوم، سرب و جیوه بر باکتری‌های غالب موجود در رآکتور منقطع متوالی (Sequencing batch reactor یا SBR) مورد بررسی قرار گرفت روش‌ها: نمونه بیومس SBR از واحد بیولوژیکی SBR تصفیه‌خانه شهرک صنعتی مورچه‌خورت اصفهان تهیه شد. پس از کشت و ایزوله باکتری‌های غالب موجود در این واحد، توالی DNA آن‌ها به وسیله تکنیک واکنش زنجیره‌ای پلیمراز (Polymerase chain reaction یا PCR) تعیین و نوع باکتری‌ها نیز مشخص گردید. پس از مواجهه این باکتری‌ها با غلظت‌های مختلفی از فلزات جیوه، کادمیوم و سرب، مقادیر 50EC آن‌ها با استفاده از آزمون زیستی Resazurin تعیین شد. یافته‌ها: چهار گونه باکتریایی «آئروموناس، سودوموناس، باسیلوس و انتروباکتر»، بیشترین تعداد را در SBR داشتند. تفاوت زیادی در حساسیت این چهار باکتری به فلزات سنگین مشاهده گردید و سودوموناس حساس‌ترین باکتری به کادمیوم و جیوه بود؛ در حالی که آئروموناس گونه حساسی به فلز سرب بود. نتیجه‌گیری: می‌توان گفت که سودوموناس و آئروموناس بیواندیکاتورهای باکتریایی مناسبی جهت ارزیابی سمیت فاضلاب‌ها و پساب‌های آلوده به فلزات سنگین می‌باشند.

Title: ارزیابی کیفیت میکروبی منابع آبی مختلف با استفاده از روش سنجش آنزیمی در مقایسه با روش متداول تخمیر چند لوله‌ای

http://hsr.mui.ac.ir/index.php/jhsr/article/view/2367

مقدمه: پایش میکروبی منابع آبی به منظور اجرای مدیریت صحیح این منابع حیاتی، ضروری می‌باشد. روش معمول برای پایش میکروبی منابع آبی، ردیابی میکروارگانیسم‏های شاخص توسط روش متداول تخمیر چند لوله‏ای است، اما امروزه روش سنجش آنزیمی به عنوان رویکرد جانشین، توسعه یافته است. هدف از انجام مطالعه حاضر، مقایسه روش سنجش آنزیمی با روش متداول تخمیر چند لوله‏ای، برای شمارش کلی‌فرم‌های کل و مدفوعی در آب‌های آشامیدنی و غیر آشامیدنی بود.  روش‌ها: در این تحقیق، 64 نمونه از منابع آبی مختلف (شرب، چشمه، چاه و قنات و پساب) برداشت گردید. ردیابی کلی‌فرم‌های کل و مدفوعی (اشرشیا کلی) در نمونه‌های فوق توسط روش متداول تخمیر چند لوله‏ای و روش سنجش آنزیمی و با استفاده از محیط کشت LMX Broth، انجام گرفت. جهت بررسي ميزان ارتباط پارامترهاي اندازه‏گيري شده توسط دو روش، از آزمون همبستگي Pearson استفاده شد. همچنین، میزان حساسیت، ویژگی و صحت روش سنجش آنزیمی برآورد گردید. یافته‌ها: یافته‌ها ارتباط بالایی را بین اندازه‌گیری کلی‌فرم کل و مدفوعی از طریق هر دو روش سنجش آنزیمی و تخمیر چند لوله‌ای نشان داد. در کل، روش سنجش آنزیمی از حساسیت، ویژگی و دقتی به ترتیب حدود 92، 95 و 94 درصد برخوردار بود. نتیجه‌گیری: با توجه به مزایای روش سنجش آنزیمی از قبیل سرعت، حساسیت و دقت به نسبت بالا و ردیابی هم‌زمان کلی‌فرم‏های کل و اشرشیاکلی، می‏توان روش سنجش آنزیمی با استفاده از LMX را به عنوان روش جایگزینی جهت روش تخمیر چند لوله‏ای به کار برد. واژه‌های کلیدی: سنجش آنزیمی؛ تخمیر چند لوله‏ای؛ کیفیت میکروبی؛ منابع آبی؛ LMX

Title: تأثیر آموزش مبتنی بر الگوی فرانظری بر ارتقای فعالیت جسمانی کارمندان جزیره ابوموسی

http://hsr.mui.ac.ir/index.php/jhsr/article/view/1981

مقدمه: ارتقای فعالیت‌های جسمانی یکی از مؤثرترین استراتژی‌ها جهت کاهش خطر بعضی از بیماری‌های غیر واگیر و مزمن است. يكي از رويكردهاي نظري براي ارتقای فعاليت جسماني، الگوي فرانظري است. هدف از انجام پژوهش حاضر، تعيين تأثير آموزش مبتنی بر الگوی فرانظری بر ارتقای فعالیت جسمانی در کارمندان جزیره ابوموسی بود. روش‌ها: در این مطالعه نیمه تجربی، کارمندان به دو گروه مداخله و شاهد تقسیم شدند و در سه مرحله قبل، 3 و 6 ماه بعد از مداخله آموزشی مورد ارزیابی قرار گرفتند. متغیرهای مورد بررسی شامل اطلاعات دموگرافیک، مراحل تغییر رفتار فعالیت جسمی، منافع و موانع درک شده و خودکارامدی نسبت به فعالیت جسمانی بود. مداخله آموزشی مبتنی بر الگوی فرانظری شامل سخنرانی، بحث گروهی، پوستر، پمفلت و فیلم آموزشی بود. داده‌ها در نرم‌افزار آماری SPSS تجزیه و تحلیل شد. یافته‌ها: قبل از مداخله آموزشی گروه مداخله و شاهد از نظر فعالیت جسمانی اختلاف معنی‌داری نداشتند. در افراد گروه مداخله، 3 ماه پس از مداخله آموزشی، فعالیت جسمانی به طور معنی‌داری افزایش یافت (05/0 < P). 6 ماه پس از مداخله آموزشی، میزان فعالیت جسمانی در گروه مداخله و شاهد اختلاف معنی‌داری نداشت (05/0 > P). نتیجه‌گیری: نتایج این مطالعه نشان داد که مداخله مبتنی بر الگوی فرانظری، می‌تواند در تغییر رفتار فعالیت جسمی کارمندان تا 3 ماه  بعد از مداخله آموزشی، مؤثر باشد. اما با گذشت زمان تغییرات ایجاد شده ممکن است کاهش یابد. بنابراین، استفاده از این الگو جهت ارتقای فعالیت جسمی، باید به طور مداوم باشد و تا رسیدن کلیه افراد به مرحله حفظ و نگهداری ادامه یابد.

Title: بررسي و تعيين شاخص‌های پرتو فرابنفش و ذرات معلق (10PM، 5/2PM و 1PM) در استان زنجان

http://hsr.mui.ac.ir/index.php/jhsr/article/view/2300

مقدمه: با توجه به اهميت روزافزون ذرات معلق و اعلام وضعيت هوا در بسياري از كشورها از نظر ميزان پرتو فرابنفش، در این مطالعه داده‌‌های ماکزیمم آلودگی ذرات معلق 10، 5/2 و 1 میکرونی که به صورت سری زمانی 1 ساعته تهیه شد و شاخص جهانی پرتو فرابنفش، به منظور پیش‌‌بینی میزان آلودگی این آلاینده‌ها مورد استفاده قرار گرفت. روش‌ها: به منظور ارایه حدود تقريبي اين شاخص در استان زنجان از تکنيک همپوشاني، نقشه ايران و نقشه پرتو فرابنفش ارایه شده توسط سازمان جهاني بهداشت براي مناطق مختلف جهان استفاده شده است. به منظور اندازه‌گيري ذرات معلق از دستگاه سنجش گرد و غبار محيطي در شهر زنجان استفاده شد که به مدت يک ساعت به صورت يک روز در ميان از اواسط مرداد ماه تا اواسط شهريور ماه اقدام به نمونه‌برداري شد. یافته‌ها: با توجه به روش تکنيک همپوشاني نقشه‌ها، مقدار شاخص پرتو فرابنفش براي استان زنجان 4 تا 5 ‌بود که مطابق با محدوده طبقه‌بندي شاخص در حد متوسط از نظر در معرض قرارگيري مي‌باشد و با داده‌های سامانه جامع اطلاعات بهداشت محیط کشور مطابقت دارد. مقدار متوسط يک ساعته ذرات معلق براي 10PM، 5/2PM و 1PM به ترتيب برابر با 95/64، 60/18 و 67/12 ميکروگرم بر متر مکعب براي شهر زنجان به دست آمد. نتیجه‌گیری: از نظر مقدار ذرات معلق در مقایسه با استانداردهای کيفيت هواي آزاد شهر زنجان شرایط مطلوب بود. در تفسیر شاخص پرتو فرابنفش می‌توان گفت که استان زنجان از نظر شاخص پرتو فرابنفش در مقایسه با معیار مورد استفاده، در وضعیت متوسط می‌باشد. با توجه به افزایش مقدار این شاخص در کشور از شمال به سمت جنوب، استان زنجان در وضعیت بهتری نسبت به استان‌های جنوبی‌تر قرار دارد.

Title: بررسی نقش ورزش و فعالیت‌های بدنی بر میزان مشارکت پرستاران در تصمیم‌گیری بالینی: مطالعه موردی در بیمارستان الزهرای (س) اصفهان

http://hsr.mui.ac.ir/index.php/jhsr/article/view/2180

مقدمه: تصميم‌‌گيري باليني جزء اساسي عملكرد حرفه‌اي پرستاران و متمايز كننده پرستاران حرفه‌اي از کارکنان مراقبتي غير حرفه‌اي است. هدف از انجام پژوهش حاضر، بررسی رابطه بین میزان پرداختن به ورزش و فعالیت‌های بدنی با میزان مشارکت پرستاران در تصمیم‌گیری بالینی در بیمارستان الزهرای (س) اصفهان بود. روش‌ها: این تحقیق از توع توصیفی- همبستگی بود و به روش پیمایشی انجام شد. جامعه آماری پژوهش را کلیه پرستاران شاغل در بیمارستان الزهرای (س) اصفهان در سال 1393 (1125 نفر) تشکیل داد. ابزار جمع‌آوری اطلاعات، پرسش‌نامه‌ تصمیم‌گیری بالینی (Participation in decision activities questionnaire‎ یا PDAQ) و پرسش‌نامه محقق ساخته فعالیت‌های بدنی بود که روایی صوری و محتوایی آن مورد تأیید 8 نفر از متخصصان رشته‌های پرستاری و مراقبت‌های بالینی و تربیت بدنی قرار گرفت. همچنین، پایایی ابزارها از طریق ضریب آلفای Cronbach به ترتیب 90/0 و 81/0 به دست آمد. یافته‌ها: بین میزان پرداختن به ورزش و فعالیت‌های بدنی با مشارکت در تصمیم‌گیری بالینی پرستاران، رابطه معنی‌داری وجود داشت (001/0 = P، 476/0 = r). همچنین، نتایج رگرسیون چندگانه برای ورزش‌های گروهی مانند والیبال، بسکتبال، بدمینتون و فوتسال با ضریب بتای 398/0 معنی‌دار بود (034/0 = P). نتیجه‌گیری: نتایج به دست آمده حاکی از تأثیر مثبت ورزش بر تصمیم‌‌گیری بالینی پرستاران و مؤلفه‌های آن همچون شناسایی مشکل برای ارزیابی راه‌حل‏های پیشنهادی و انتخاب بهترین گزینه می‌باشد؛ به طوری که با انجام ورزش، پرستاران قادر به اتخاذ تصمیم‌های مناسب‌تر و انتخاب راه‌حل‌های صحیح‌تری خواهند بود.

Title: شیوع اختلالات رفتاری در کودکان ایران

http://hsr.mui.ac.ir/index.php/jhsr/article/view/2222

مقدمه: اختلالات رفتاري در كودكان، با كاهش يادگيري و پيشرفت تحصيلي در دوران كودكي و بروز اختلالات رفتاري در بزرگسالي ارتباط دارد. هدف از انجام مطالعه حاضر، تعيين شيوع اختلالات رفتاري در کودکان ايران بود. روش‌ها: در اين مطالعه توصيفي- مقطعي، تمام دانش‌آموزان سال اول ابتدايي، اول راهنمايي و اول دبيرستان در سراسر ايران، توسط دانشگاه‌هاي علوم پزشكي كشور از نظر اختلالات رفتاري غربالگري شدند. براي جمع‌آوري داده‌ها، از پرسش‌نامه Rutter‎ فرم والدين استفاده شد. یافته‌ها: 42/1 درصد از كودكان به اختلالات رفتاري مبتلا بودند. شيوع اختلالات رفتاري در پسران و دختران مساوي بود (5/1 درصد). همچنین، شيوع این اختلالات در كودكان شهری، 6/1 درصد و در كودكان روستايي، 4/1 درصد به دست آمد. شيوع اختلالات رفتاري در دانش‌آموزان سال اول ابتدايي (47/2 درصد) نسبت به دو مقطع ديگر بيشتر بود. نتیجه‌گیری: شيوع اختلالات رفتاري در كودكان به ويژه در گروه سني سال اول ابتدايي، نگران كننده است و اهميت بيماريابي دقيق با برنامه‌هاي غربالگري گسترده و برنامه‌ریزی مناسب برای پیشگیری را نشان می‌دهد.

Title: ارزیابی آگاهی، نگرش و عملکرد زنان باردار شهر زاهدان در زمینه عفونت‌های ادراری بر مبنای ساختارهای مدل اعتقاد بهداشتی

http://hsr.mui.ac.ir/index.php/jhsr/article/view/1982

مقدمه: عفونت ادراری یکی از شایع‌ترین عفونت‌های زنان به شمار می‌رود و در دوران بارداری به علت تغییراتی که در بدن رخ می‌دهد، احتمال ابتلا به آن بیشتر می‌شود و به علت عوارض بالقوه‌ای که بر مادر و جنین دارد، بسیار مهم است. مطالعه حاضر با هدف تعیین آگاهی و نگرش زنان باردار درباره عفونت‌های ادراری بر اساس مدل اعتقاد بهداشتی انجام شد. روش‌ها: این مطالعه توصیفی- تحلیلی بر روی 140 زن باردار مراجعه کننده به مراکز بهداشتی- درمانی شهر زاهدان انجام گرفت. ابزار جمع‌آوری اطلاعات، پرسش‌نامه‌ای چند بخشی شامل اطلاعات فردی، آگاهی، سؤالات اجزای مدل (ادراک) و رفتار بود. روایی پرسش‌‌نامه با استفاده از نظر استادان (81 درصد) و پایایی آن با محاسبه ضریب Cronbach¢s alpha (67 درصد) تأیید شد. داده‌ها با استفاده از آزمون‌های Pearson، Independent t و ANOVA در نرم‌افزار آماری SPSS و با سطح اطمینان 95 درصد مورد تجزیه و تحلیل قرار گرفت. یافته‌ها: میانگین سنی افراد مورد مطالعه 00/4 ± 55/22 سال بود و 3/64 درصد از آن‌ها تحصیلات کمتر از 9 سال داشتند. نمره آگاهی فقط در 9/24 درصد افراد خوب برآورد شد. نمره حساسیت درک شده در 4/40 درصد ، شدت درک شده در 7/49 درصد، موانع درک شده در 1/56 درصد، منافع درک شده در 0/61 درصد و رفتار در 0/38 درصد از شرکت کنندگان، خوب ارزیابی گردید. بین افراد شاغل و خانه‌دار از نظر آگاهی اختلاف معنی‌داری وجود داشت و بین نمره آگاهی با نمرات همه اجزای مدل همبستگی مثبت معنی‌داری مشاهده شد. نتیجه‌گیری: بر اساس نتایج به دست آمده، میزان آگاهی مشارکت کنندگان در زمینه عفونت ادراری ضعیف است و نگرش و رفتار آنان نیز در سطح متوسطی قرار دارد. بنابراین، پیشنهاد می‌شود که جلسات و کلاس‌های آموزشی با هدف ارتقای آگاهی، نگرش و رفتار زنان در زمینه عفونت ادراری بر اساس مدل اعتقاد بهداشتی که چارچوب و خط‌مشی را مشخص می‌کند، برگزار گردد.

Title: امکان‌سنجی استفاده از لوله امپدانس با دو میکروفن و اندازه‌گیری ضریب جذب صوتی برخی از مواد جاذب ایرانی به روش تابع انتقال

http://hsr.mui.ac.ir/index.php/jhsr/article/view/2333

مقدمه: صدا یکی از مهم‌ترین عوامل تهدید کننده سلامت شاغلان می‌باشد. مواد جاذب روش مؤثری برای کنترل صدا است که لوله امپدانس با دو میکروفن جهت تعیین ضرایب جذب آن‌ها کاربرد دارد و دقت و کارایی آن باید به صورت دوره‌ای تست شود. بنابراین، پژوهش حاضر با هدف امکان‌سنجی عملکرد لوله امپدانس و تعیین ضریب جذب برخی از مواد انجام شد. روش‌ها: پس از ساخت لوله امپدانس و اطمینان از کارایی آن بر اساس مقایسه نمودار فوم استاندارد با نمودار اندازه‌گیری شده، ضرایب جذب چهار نمونه شامل پشم شیشه (چگالی  15 کیلوگرم بر مترمکعب)، پشم معدنی (چگالی 40 کیلوگرم بر متر مکعب)، چوب پنبه (چگالی 390 کیلوگرم بر متر مکعب) و لاستیک (چگالی 1100 کیلوگرم بر متر مکعب) در دو ضخامت مختلف ارزیابی گردید. همچنین، ضرایب جذب پشم شیشه و پشم معدنی با مقادیر ضرایب جذب ذکر شده مربوط به ‏این دو نمونه در خارج از ایران (به عنوان مرجع)، با ضخامت و چگالی یکسان مقایسه شد. اندازه‌گیری هر نمونه توسط نرم‌افزار VA-Lab4 انجام گردید و در نهایت، نتایج به صورت نمودار نمایش داده شد. یافته‌ها: نتایج دقت و کارایی نشان داد که لوله امپدانس با ضریب همبستگی 98/0 قابل استفاده بود. همچنین، نمونه‌های پشم معدنی و پشم شیشه بیشترین ضریب جذب و لاستیک و چوب پنبه کمترین ضریب را داشتند. مقایسه پشم شیشه با مقادیر مرجع نشان داد که در هر دو با افزایش فرکانس، میزان جذب نیز افزایش یافت که البته مقادیر ضرایب جذب این دو نمونه متفاوت بود. علاوه بر این، در نمونه پشم معدنی اندازه‌گیری شده با ضخامت 50 میلی‌متر نسبت به ضخامت کمتر، در همه فرکانس‌ها به جز محدوده 1250 تا 3200 هرتز، جذب بیشتری مشاهده شد؛ در حالی که پشم معدنی مرجع با ضخامت بیشتر در فرکانس‌های 2000 و 5000 هرتز، ضریب جذب کمتری را نشان داد. نتیجه‌گیری: با توجه به نتایج، می‌توان ضریب جذب همه مواد و مصالح را با بالاترین دقت توسط لوله امپدانس با دو میکروفن تعیین نمود تا به نتایج مطلوبی از روش‌های کنترلی آلودگی صوتی با استفاده از جاذب‌های صوتی در محیط و صنعت دست یافت.
